# Brain and Behavior Plasticity: From Fundamental Science to Health Outcomes

**DOI:** 10.1155/2015/985790

**Published:** 2015-09-01

**Authors:** Keh-chung Lin, Steven L. Wolf, Chetwyn Chan, Ching-yi Wu, Ching-Po Lin

**Affiliations:** ^1^School of Occupational Therapy, National Taiwan University College of Medicine, Taipei, Taiwan; ^2^Division of Occupational Therapy, Department of Rehabilitation Medicine, National Taiwan University Hospital, Taipei, Taiwan; ^3^Department of Rehabilitation Medicine, Emory University School of Medicine, Atlanta, GA 30322, USA; ^4^Department of Rehabilitation Sciences, The Hong Kong Polytechnic University, Hong Kong; ^5^Department of Occupational Therapy and Graduate Institute of Behavioral Sciences, Chang Gung University, Taoyuan, Taiwan; ^6^Institute of Brain Science, National Yang-Ming University, Taipei, Taiwan

Human brains are highly plastic throughout the life­span, even after brain injuries. The possible modulation of functions due to neural plasticity in various brain regions contributes to the learning and adaptation of unlearnt as well as new behaviors. Over the past decade, research on brain-behavior relationships has grown exponentially. In addition to behavioral assessments, electrophysiology and neuroimaging methods have been widely used to examine the neural networks, structural and functional abnormalities, and the effects of the therapeutic training in aged adults or individuals with neurological disorders. The five contributions to this special issue address the relationships between brain and behavior and have implications for present and future research and practice in neurorehabilitation.

Data presented in the article titled “An Influence of Birth Weight, Gestational Age, and Apgar Score on Pattern Visual Evoked Potentials in Children with History of Prematurity” by M. Michalczuk et al. suggests that low birth weight, early gestational age, and poor baseline output are possible predictors for the development rate of brain function. The article titled “Age-Related Reduced Somatosensory Gating is Associated with Altered Alpha Frequency Desynchronization” by C.-H. Cheng et al. attempts to elucidate the neural mechanisms of age-related sensory gating decline in the somatosensory system. By using a paired-pulse protocol and time frequency analysis, the authors found that insufficient stimulus 1 induced alpha oscillations lead to a less-suppressed stimulus 2 evoked response.

The article titled “Coincidence Anticipation Timing Performance during an Acute Bout of Brisk Walking in Older Adults: Effects of Stimulus Speed” by M. J. Duncan et al. reported an age-related decline in anticipation timing performance when performing dual tasks. They have also reported that the stimulus speed of the secondary task plays an important role in the performance of older adults. Data from the article titled “Neural Plastic Effects on Cognitive Training on Aging Brain” by N. T. Y. Leung et al. suggested the presence of an experience-dependent neural plasticity following a thirteen-week training program of attention and working memory in older adults. This work also indicates that initial cognitive status may not limit the potential of neural plasticity at an older age. The article titled “Restoration of Central Programmed Movement Pattern by Temporal Electrical Stimulation-Assisted Training in Patients with Spinal Cerebellar Atrophy” by Y. Z. Huang et al. reported a novel 4-week training program developed to correct the aberrant triphasic EMG patterns in these patients resulting in restoration of facilitation of antagonist muscle activity.

The articles published in this special issue provide new insights into the neural plasticity in terms of the brain-and-behavior relations and contribute to extension of research on neurobehavioral rehabilitation. Further research is needed to verify the value of the knowledge derived from this collection of scientific endeavors.

*Keh-chung Lin* *Keh-chung Lin* *Steven L. Wolf* *Steven L. Wolf* *Chetwyn Chan* *Chetwyn Chan* *Ching-yi Wu* *Ching-yi Wu* *Ching-Po Lin* *Ching-Po Lin*

